# White matter integrity in individuals at ultra-high risk for psychosis: a systematic review and discussion of the role of polyunsaturated fatty acids

**DOI:** 10.1186/s12888-016-0932-4

**Published:** 2016-08-11

**Authors:** Nandita Vijayakumar, Cali Bartholomeusz, Thomas Whitford, Daniel F. Hermens, Barnaby Nelson, Simon Rice, Sarah Whittle, Christos Pantelis, Patrick McGorry, Miriam R. Schäfer, G. Paul Amminger

**Affiliations:** 1 Orygen, The National Centre of Excellence in Youth Mental Health, The University of Melbourne, 35 Poplar Road, Parkville, Victoria 3052 Australia; 2 Department of Psychology, University of Oregon, Eugene, USA; 3 School of Psychology, UNSW Australia, Sydney, Australia; 4 Brain & Mind Research Institute, Central Clinical School, University of Sydney, Sydney, Australia; 5 Melbourne Neuropsychiatry Centre, The University of Melbourne, Melbourne, Australia

**Keywords:** Ultra-high risk, Psychosis, White matter, Polyunsaturated fatty acids, Youth

## Abstract

**Background:**

Schizophrenia is thought to be a neurodevelopmental disorder with pathophysiological processes beginning in the brain prior to the emergence of clinical symptoms. Recent evidence from neuroimaging studies using techniques such as diffusion tensor imaging has identified white matter abnormalities that are suggestive of disrupted brain myelination and neuronal connectivity. Identifying whether such effects exist in individuals at high risk for developing psychosis may help with prevention and early intervention strategies. In addition, there is preliminary evidence for a role of lipid biology in the onset of psychosis, along with well-established evidence of its role in myelination of white matter tracts. As such, this article synthesises the literature on polyunsaturated fatty acids (PUFAs) in myelination and schizophrenia, hypothesizing that white matter abnormalities may potentially mediate the relationship between PUFAs and schizophrenia.

**Methods:**

Diffusion tensor imaging studies were identified through a systematic search of existing literature. Studies examined white matter integrity in ultra-high risk (UHR) samples, as assessed using structured diagnostic interviews. Data was extracted and summarised as a narrative review.

**Results:**

Twelve studies met inclusion criteria, and findings identified reduced fractional anisotropy and higher diffusivity. Although the exact location of abnormalities remains uncertain, fronto-temporal and fronto-limbic connections, including the superior longitudinal and uncinate fasiculus, cingulum, and corpus callosum appear to be implicated. Because of preliminary evidence suggesting lipid biology may be relevant for the onset of psychosis, a discussion is provided of the role of polyunsaturated fatty acids (PUFAs) in myelination and risk for psychosis.

**Conclusions:**

While the function of PUFAs in myelination is well-established, there is growing evidence of reduced PUFA concentration in UHR samples, highlighting the need for research to examine the relationship between PUFA and white matter integrity in high-risk samples and age-matched healthy controls. Such investigations will help to better understand the pathophysiology of the disorder, and potentially assist in the development of novel treatment and early intervention strategies.

## Background

Schizophrenia is often a chronic disorder, characterised by delusions, hallucinations, blunted affect and cognitive impairment [1, 2], which commonly presents during late adolescence or early adulthood [3]. Though it has long been postulated to be a disorder of neurodevelopment with pathophysiological processes beginning in the brain prior to the onset of clinical symptoms [1, 2], the advent of neuroimaging techniques has been instrumental in probing these neurobiological processes. While these techniques have provided unique insights into various aspects of brain morphometry and function, of particular relevance to psychosis is diffusion tensor imaging (DTI) studies on white matter integrity in fibre tracts connecting brain regions. We review this research within samples at ultra-high risk (UHR) for psychosis, in order to identify white matter abnormalities that may be implicated in the *development* of the illness. There is also a substantial body of evidence for lowered cell membrane polyunsaturated fatty acids (PUFA) concentrations in schizophrenia, which compose the myelin sheath surrounding white-matter tracts. Therefore, we end the review with a discussion about the role of PUFAs in myelination and schizophrenia, hypothesizing that white matter abnormalities may potentially mediate the relationship between PUFAs and schizophrenia. We also highlight the need to examine these relationships in further detail among UHR samples to better understand the pathophysiology of the disorder, and potentially assist in the development of novel treatment and early intervention strategies.

DTI measures the diffusion of water molecules through tissues, and provides a measure of net directionality and magnitude (diffusivity). The net directionality of diffusion, indexed by fractional anisotropy (FA), is the most common index of white matter integrity. Lower FA has been associated with reduced axonal caliber and packing density, myelin pathology, as well as less coherent fibres or crossing fibres within a voxel [4]. Mean diffusivity (MD) is another frequently used index that quantifies overall diffusion within a particular voxel, with higher values suggestive of disrupted axonal integrity [5]. Research on normative developmental patterns of white matter integrity has identified significant increases in FA and decreases in diffusivities during childhood and adolescence across all the major fibre tracts [6]. While most of these changes are complete by the end of adolescence, some association tracts (i.e. inferior and superior longitudinal and fronto-occipital fasciculi) continue to develop during early adulthood [7]. It is particularly important to consider these normative developmental trajectories when interpreting white matter integrity in UHR samples, given that most of these individuals are adolescents.

### White matter integrity in schizophrenia

DTI studies have provided extensive evidence for lowered white matter integrity across the brain in schizophrenia, including the uncinate fasiculus and fornix, fronto-occipital fasiculus, arcuate fasiculus, anterior commissure, cingulum bundle and corticospinal tract [for review, see [8, 9]]. However, it is uncertain whether these white matter abnormalities are intrinsic to schizophrenia, given that these studies are often confounded by multiple factors, including chronicity of the illness, treatment with antipsychotic medication [10, 11], sex and age of participants [12], and even heavy cigarette smoking [13].

Longitudinal research on individuals at UHR for psychosis enables assessment prior to, during and following the onset of illness. These individuals represent a ‘clinical high-risk’ group, who present with sub-threshold or brief psychotic symptoms, may have trait factors such as a genetic risk for the disorder, and exhibit a significant decline in functioning [14]. This prodromal phase is associated with enhanced risk for development of schizophrenia compared to the general population or to those at genetic risk for the illness, with transition rates ranging from 18 % after six months to 35 % after a ten year period [15, 16]. Thus, by following these individuals over time, it is possible to identify factors that may predict transition to psychotic illnesses, as well as better understand the neurobiological processes underlying this progression [17, 18], including white matter abnormalities during transition to psychosis [19]. This information may help identify early intervention and preventative measures that may halt the progression from sub-threshold symptoms to fully-fledged illness. Below, we provide a *systematic review* of DTI findings in individuals at UHR for psychosis.

## Methods

**Fig. 1:**
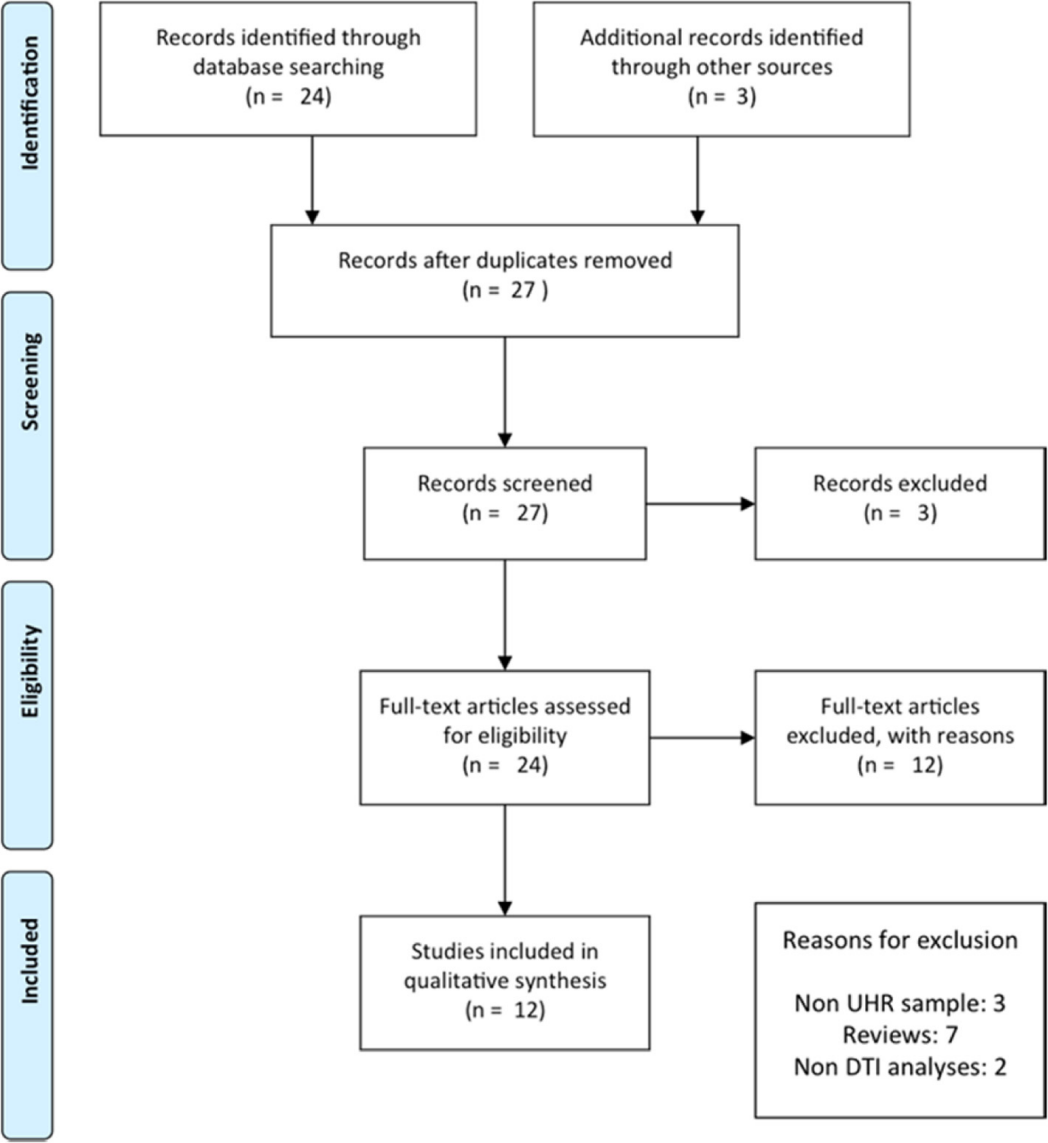
PRISMA 2009 Flow Diagram

We followed the Preferred Reporting Items for Systematic Reviews and Meta-Analyses’ (PRISMA) guidelines. An electronic search was conducted in PubMed and Medline, using the key words *high risk*, *psychosis*, *diffusion tensor imaging* and *white matter,* to identify studies published in this field to date (July 2015). Inclusion criteria for the review were: i. employed DTI, ii. included subjects at ultra high risk for psychosis, assessed using structured diagnostic interviews (such as the Structured Interview for Prodromal Syndromes [20] or the Comprehensive Assessment of At-Risk Mental States [21]), iii. compared these participants to a healthy control group on DTI measures, and iv. were written in English. The reference lists of identified articles were also searched for further relevant articles. Figure 1 summarizes the search strategy used for selecting studies [identification, screening, eligibility, inclusion process] in the present review. We excluded studies that were classified as high risk based only on familial risk alone. In total, 12 studies were identified that fit the above-mentioned criteria (See Table 1 for details).

**Table 1:** Diffusion tensor imaging studies on ultra-high risk samples

Reference	Population	UHR assessment	DTI parameters^a^	Technique	Major Findings
Bloemen et al., [30]	27 UHR-N (18 M, mean age = 18.9), 10 UHR-P (8 M, mean age = 20.7), 10 HC (8 M, mean age = 22.7)	SIPS	3 T, EPI, 48 continuous 3 mm slices, 2x2x3mm	VBA, whole brain	UHR-P vs. HC: ↓ FA in bilateral medial frontal lobes.
					UHR-P vs. UHR-N: ↓ FA lateral to right putamen and left superior temporal lobe,↑FA in left medial temporal lobe.
					Positive PANSS negatively correlated with FA in left medial temporal lobe in UHR-P group, and right superior temporal lobe in total UHR group.
Carletti et a., [31]	Baseline: 32 UHR (19 M, mean age = 23.4), 14 EOS (14 M, mean age = 25.9), 32 HC (27 M, mean age = 25.9)	CAARMS	1.5 T, EPI, 60 contiguous 2.5 mm axial slices, 1.875x1.875x2.5 mm	VBA, whole brain	Baseline: FA was lowest in EOS, highest in HC and intermediate in UHR group. Clusters comprised in areas corresponding to CC, left ILF & SLF, left IFOF and cortico-subcortical pathways.
	Follow-up: 22 UHR (5 UHR-P, 17 UHR-N) subjects (11 M, mean age = 26.6), 8 NC (17 M, mean age = 29.6)				Longitudinal UHR-P vs. UHR-N: ↓ FA over time in left frontal white matter, CC, SCR and SFOF. But no significant within group change.
Bernard et al., [29]	26UHR (20 M, mean age = 18.5), 21 HC (15 M, mean age = 17.77)	SIPS	3 T, EPI, GRAPPA parallel imaging factor 2, 72 slices, 2x2x2mm	TBSS, ROI = thalamic-hippocampal tract	UHR vs. HC: Significant group*time interaction. Controls FA ↑ over 12 months, while UHR FA ↓ over time (but no significant main effect of time).
	Follow-up: 15UHR, 15 HC				
Clemm von Hohenberg et al., [25]	28 UHR (18 M, mean age = 20.6), 34 HC (18 F, mean age = 20.4)	SOPS	3 T, EPI, 75 contiguous axial 2 mm slices, 2x2x2mm	TBSS, whole brain	UHR vs. HC: MD ↑ in several right hemisphere clusters (most notably SLF, posterior corona radiata, and CC). RD ↑ posterior parietal lobe.
Epstein et al. [24]	21 UHR (18 M, mean age = 16.1), 55 EOS (31 M, mean age = 16.9), 55 HC (27 M, mean age = 16.5), 31 cannabis use (non-psychotic, 22 M, mean age = 17.6).	SIPS	3 T, EPI, 64 sagittal 2 mm slices, 2x2x2mm	Fiber tracking, ROIs = CB, SLF, CST, ILF, IFOF, and UF	EOS and UHR vs. HC and cannabis: FA ↓ bilateral CST
					EOS and UHR vs. HC: FA ↓ left ILF and IFOF
Karlsgodt et al., [27]	36 UHR (27 M, mean age = 17.0), 25 HC (12 M, mean age = 18.0)	SIPS	1.5 T, EPI, 75 contiguous 2 mm AC-PC interleaved slices, 2x2x2mm	TBSS, ROIs = UF, AF, CB, ILF, MTL, ATR	UHR vs. HC: FA ↓ SLF at baseline. Did not increase FA with age in MTL and ILF.
					FA ↓ MTL and ILF at baseline predicted reductions in functional outcomes in UHR.
Katagiri et al., [32]	16HC (8 M, mean age = 23.2), 11 UHR-NN(3 M, mean age = 24.2), 23 UHR-NA (6 M, mean age = 23.4), 7 UHR-P (1 M, mean age = 20.7)	SIPS	1.5 T, EPI, 30 axial slices, 1.02 × 1.02 × 5 mm	TBSS, whole brain for baseline analyses, followed by ROI (CC) for longitudinal analyses	Baseline: UHR vs. HC: ↓ FA in CC. Result also present in UHR-N vs. HC. Longitudinal: UHR-N improvements in positive symptoms at follow-up, which correlated with increased FA in the CC.
	Follow-up: same groups as above				
Mittal et al., [28]	33UHR (20 M, mean age = 18.5), 35 HC (15 M, mean age = 17.77)	SIPS	3 T, EPI, GRAPPA parallel imaging factor 2, 72 slices, 2x2x2mm	TBSS, ROI = SCP	UHR vs. HC: No group differences in baseline SCPs. Controls FA ↑ over 12 months, while UHR FA ↓ over time.
	Follow-up: 15UHR, 15 HC				
Peters et al., [22]	10 UHR (mean age = 21.2), 10 EOS (mean age = 21.6) and 10 HC (mean age = 21.1). All male sample.	SIPS	3 T, EPI, 48 continuous (para)transversal slices, 3x3.5x2.2 mm	Fiber tracking, ROIs = UF, AF, CB, CC	No group differences
Peters et al., [23]	Same subjects as Peters et al. (2008)	SIPS	As above	VBA, whole brain	UHR vs. HC: FA ↓ bilateral superior and middle frontal
					EOS vs. HC: FA ↓ bilateral temporal & parietal, and left frontal
Peters et al., [26]	10 UHR-N (mean age = 21.2), 7 UHR-P (mean age = 22.6), 10 HC (mean age = 21.1). All male sample.	SIPS	As above	Fiber tracking, ROIs = UF, AF, CB, CC	No group differences
Petersson-Yeo et al., [33]	19 (12 M) pairs EOS (mean age = 24.37) vs. HC (mean age = 24.89)	CAARMS	3 T, EPI, 60 contiguous axial 2.4 mm slices	TBSS, whole brain	FA differentiated UHR and EOS from HC. Pattern of findings were widely and diffusely spread, with no clear concentration of regions.
					FA did not differentiate UHR and EOS.
	19 (9 M) pairs UHR (mean age = 22.42) vs. HC (mean age = 23.32)				
	15 (9 M) pairs UHR (mean age = 23.2) vs. EOS (mean age = 23.2)				

^a^DTI parameters: field strength, acquisition technique, slice number/thickness/direction, voxel dimensions AC-PC: anterior commissure - posterior commissure; AF = arcuate fasiculus; ATR = anterior thalamic radiation; CAARMS: Comprehensive Assessment of At-Risk Mental States; CB = cingulate bundle; CC = corpus callosum; CST = cortiospinal tract; EOS = early onset schizophrenia; EPI: echo planar imaging; GRAPPA = generalized autocalibrating partially parallel acquisitions; HC = healthy controls; IFOF = inferior fronto-occipital fasiculus; ILF = inferior longitudinal fasiculus; ROI = region of interest; SCP = Superior cerebellar peduncle; SCR = superior corona radiata; SFOF = superior frontal occipital fasciculus; SIPS: Structured Interview for Prodromal Syndromes; SLF = superior longitudinal fasiculus; SOPS: Scale of Prodromal Symptoms; TBSS = tract based spatial statistics; UF = uncinate fasiculus; UHR = ultra high risk for schizophrenia; UHR-P = ultra-high risk subjects who transitioned to psychosis; UHR-N = ultra-high risk subjects who did not transition to psychosis; UHR-NA = ultra-high risk subjects who did not transition to psychosis and were prescribed antipsychotic mediation; UHR-NN = ultra-high risk subjects who did not transition to psychosis and were not prescribed antipsychotic mediation; VBA = voxel based analyses

## Results and discussion

### DTI studies of individuals at ultra high risk for psychosis

Peters and colleagues [22] conducted the first DTI study to investigate white matter abnormalities in a UHR sample, and failed to identify any differences in FA within a priori white matter tracts using fibretracking (including the uncinate and arcuate fasciculus, anterior and dorsal cingulum, sub-divisions of the corpus callosum) between 10 male UHR subjects, 10 with recent-onset schizophrenia and 10 healthy controls. However, a subsequent study on the same sample using whole brain voxel-wise analysis identified significantly reduced FA in UHR patients compared to controls within the white matter of the right superior frontal lobe and left middle frontal lobe [23]. While those with schizophrenia were found to have significantly reduced FA in the left frontal and bilateral temporal and parietal regions compared to controls, no significant differences were identified between UHR and schizophrenia patients. Similarly, Epstein et al. [24] found lowered FA in the bilateral corticospinal tract, left inferior longitudinal fasciculus and left inferior fronto-occipital fasciculus in UHR adolescents and those with schizophrenia compared to healthy controls, though no significant differences were identified between the two groups. A recent study that investigated whole brain differences in DTI measures also identified a trend towards reduced FA in UHR [25]. Stronger effects were identified in relation to MD, with significantly increased levels [reflecting a reduction in white matter integrity] present in the UHR group in the superior longitudinal fasiculus, corona radiata and corpus callosum.

While the aforementioned studies have identified white matter differences in a range of white matter fasciculi, they did not examine transition to psychosis within UHR samples. As mentioned earlier, while some of these UHR individuals go on to develop full-threshold psychosis, others have resolution of symptoms and some have persistent non-psychotic disorders (i.e. low-level sub-threshold symptoms). Therefore, it is important to look at longitudinal clinical and functional outcomes within UHR populations.

Among the first of the few studies to prospectively follow UHR subjects, Peters et al. [26] failed to identify any baseline differences in FA for a priori defined white matter tracts [uncinate and arcuate fasciculi, anterior and dorsal cingulate, and subdivisions of the corpus callosum] in UHR subjects who transitioned to psychosis (UHR-P, *N* = 7), those who did not transition to psychosis (UHR-N, *N* = 10), and healthy controls (*N* = 10). However, it should be noted that the small sample size in this study may have led to Type II error. In comparison, Karlsgodt et al.’s [27] region of interest study of 36 UHR subjects and 25 controls found lower baseline FA in the UHR group within the superior longitudinal fasiculus. Cross-sectional analysis of age-effects revealed that the UHR group did not exhibit the expected age-related increase in FA within the medial temporal lobe. Lower FA in the medial temporal lobe and inferior longitudinal fasiculus was also found to predict lower functioning within the UHR group at 15-month follow-up. Further, there was a trend towards lower FA in the medial temporal lobe in UHR-P (17 %) compared to UHR-N subjects.

Similarly, Mittal and colleagues’ [28] study of the cerebellar-thalamic tract did not identify any significant differences at baseline between UHR subjects (*N* = 33) and controls (*N* = 35). However, 12-month follow-up of a subset of participants [15 UHR, 15 controls] found that UHR subjects exhibited a significant reduction in FA over time compared to healthy controls, resulting in significantly lower FA at follow-up. Although the study did not examine transition to psychosis, they did find that positive and negative symptoms declined over time in UHR subjects. A subsequent study by the researchers identified a similar group × time interaction within the thalamic-hippocampal tract [29]. Specifically, UHR subjects (*N* = 26) exhibited a significant reduction in FA over time in comparison to healthy controls (*N* = 21). This was driven by a pattern of increased FA in controls over the 12-month follow-up period and reduced FA in UHR subjects, although main effect of time was not significant within either group.

Only three longitudinal studies have used a whole brain approach to examine FA differences to date. The first of these studies by Bloemen and colleagues [30] combined baseline neuroimaging assessments with clinical follow-up of UHR individuals at 24 months to ascertain transition to psychosis. They identified lower baseline FA in UHR-P (*N* = 10) compared to controls (*N* = 10) in the medial frontal lobes bilaterally, approximate to the left anterior thalamic radiation and inferior fronto-occipital fasciculus. Lower FA was also identified in white matter lateral to the right putamen and left superior temporal lobe in UHR-P compared to UHR-N (*N* = 27) groups. Interestingly, the same group comparison identified higher FA in those who transitioned within the left white matter of the medial temporal lobe. In addition, positive symptoms were negatively correlated with FA in the medial temporal lobe within those who transitioned, and were also negatively correlated with FA in the right superior temporal lobe within the overall UHR group.

Carletti et al. [31] were the first to investigate longitudinal differences in white matter, having neuroimaging follow-up of UHR subjects and healthy controls after 28 months, along with baseline scans of first episode psychosis patients. They found that first episode patients exhibited widespread reductions in FA, as well as increased radial and axial diffusivities, compared to controls at baseline. The UHR group was found to lie in between these two groups on various DTI indices, including within the parietal region, encompassing the SLF, corpus callosum, and inferior fronto-occipital fasciculus. However post-hoc analyses failed to identify any significant differences between UHR and control groups. Longitudinal follow-up revealed that UHR-P subjects (*N* = 5) exhibited a progressive reduction in FA in left frontal white matter, including the anterior limb of the internal capsule, corpus callosum, superior corona radiata and superior frontal occipital fasciculus in comparison to UHR-N subjects (*N* = 17). However, neither group exhibited significant within-group change, nor were any significant differences in comparison to controls reported. The only other study to longitudinally examine white matter changes identified significantly lowered FA within the corpus callosum in UHR subjects compared to healthy controls at baseline [32]. They were unable to examine longitudinal FA change in UHR subjects who transitioned compared to those who did not transition at 12-month follow-up due to small sample size of the former group (*N* = 4). However, they did identify a significant negative correlation between change in positive symptoms and change in FA within the same region in UHR-N subjects. This effect remained significant after accounting for age, sex and antipsychotic use. The authors argued that preservation of white matter may therefore reduce the risk of developing psychosis, thus acting as a protective neurobiological factor.

A recent study by Pettersson-Yeo et al. [33] used machine learning to examine the ability of structural magnetic resonance imaging (MRI) and functional magnetic resonance imaging (fMRI), DTI, genetic and cognitive data to differentiate between UHR, first episode patients and healthy controls at the single-subject level. They found that first episode patients were identifiable at the individual level using a range of biological and cognitive measures. Specifically, the algorithm was able to accurately classify first episode patients in comparison to controls using genotype (68 %), DTI (66 %), fMRI (66 %) and cognitive data (74 %). It was also able to differentiate first episode and UHR patients using structural MRI (77 %), fMRI (73 %) and cognitive data (67 %). In comparison, only structural MRI and DTI discriminated UHR from control participants, with an accuracy of 68 % and 66 %, respectively. These findings support the notion that white matter integrity is implicated prior to the onset of psychosis and is a sensitive measure of risk for transition.

Although limited in number, these studies provide some evidence for lower FA and higher MD in UHR subjects who transition to psychosis, relative to UHR individuals who do not transition, and healthy controls (refer to Table 1 for an overview). While the exact pattern of white matter differences is not consistent across studies, frontal, fronto-temporal and fronto-limbic connections, including the superior longitudinal and uncinate fasiculus, cingulum, and corpus callosum appear to be implicated. These findings in UHR samples suggest that white matter abnormalities may play an important role in the *development* of psychotic disorders, although further longitudinal research is needed that examines within-subject change in FA along with progression to psychosis. In addition, the small sample size of many studies to date, along with variations in DTI analytic methods, may be partly responsible for the inconsistencies in findings. Along this line, future meta-analyses may have help clarify the exact location and nature of white matter abnormalities in high-risk samples.

### White matter and polyunsaturated fatty acids

There is evidence to suggest that the observed changes in DTI indices of white matter integrity are due, at least in part, to damage to the myelin sheath that insulates axons and transmits electric signals between nerves in schizophrenia patients [34, 35]. The myelin sheath is formed by the membrane of oligodendrocytes and consists of approximately 70 % lipids, with phospholipids and cholesterol accounting for the largest proportion of membrane lipids in mammals [36, 37]. PUFAs are important components of the phospholipid layers in all cell membranes, including oligodendrocytes. Arachidonic acid (AA, C20:4n-6), docosahexaenoic acid (DHA, C22:6n-3) and nervonic acid (NA, C24:1n-9) are among the most important fatty acids in the nervous system. They impact on neuronal functioning through two main mechanisms. Firstly, they play a role in maintaining membrane structure, and modulating the function of membrane receptors, ion channels and enzymes [38]. Secondly, they are a source of precursors for eicosanoids, including prostaglandins, thromboxanes and leukotrienes, which play an important role in immune and inflammatory responses, and also act as second messengers in intra- and inter-cellular signal transduction [38].

PUFAs are acquired through dietary intake and the amount of intake affects the rate of phospholipid synthesis, which in turn impacts on the quantity and quality of membrane phospholipids [39]. If PUFAs are not available during myelin synthesis, there is disruption resulting in amyelination or dysmyelination. Such myelin changes have been identified in rats with PUFA deficiency [40], and dietary supplementation of omega-3 fatty acids in rats has been found to simulate the expression of myelin proteins in the brain [41]. In humans, dietary supplementation of PUFAs has been found to reduce the occurrence of white matter abnormalities in the elderly [42], and the exacerbation rate and disability in patients with multiple sclerosis [43]. Apart from dietary intake, genetic factors also impact the synthesis of PUFAs. The human-specific haplotype of fatty-acid desaturase (FADS) genes has been associated with decreased blood levels of DHA and AA (suggestive of lower biosynthesis of PUFAs), as well as decreased white matter development from childhood to adulthood [44].

Peters and colleagues [45] report the only study to date examining the relationship between myelination and PUFAs in healthy individuals using DTI. They investigated whether white matter integrity, using DTI analysis, was associated with blood levels of PUFA concentrations and the activity of phospholipase A_2_ (the main enzyme regulating membrane PUFA metabolism) in 9 to 20 year olds. Interestingly, they found that lower blood PUFA concentrations were related to higher FA in the corticospinal tract, anterior thalamic radiation, inferior fronto-occipital fasiculus and cingulum. In addition, lower blood PUFA concentrations were related to lower RD in the same regions, as well as the uncinate, superior and inferior longitudinal fasiculi. The authors argue that active white matter myelination during adolescence may require more vigorous consumption of PUFAs from peripheral membranes, given the continued white matter changes that have been identified during this period [46]. Hence while these findings highlight a relationship between blood PUFA concentrations and DTI-indices of white matter integrity, it remains uncertain whether the same direction of association would be present following this developmental period. There is a need for further research across different ages, ideally using longitudinal research to examine potential developmental effects and better understand these complex associations.

### Polyunsaturated fatty acids and white matter in schizophrenia

Given the relationship between PUFAs and myelination, we propose that white matter integrity may mediate the relationship between PUFAs and risk for schizophrenia. There is a substantial body of evidence for lowered blood PUFA concentrations in schizophrenia. These include reductions in blood levels of DHA, AA, NA, docosapentaenoic acid (DPA) and linoleic acid (LA) in patients [47–49], including medication-naïve patients [50, 51]. Similar findings have also been identified in postmortem studies [52]. Consistent with these findings, there is evidence that omega-3 PUFA dietary supplementation may prevent the transition from UHR to psychosis, with Amminger et al.’s [53] placebo-controlled clinical trial finding that 12 weeks of PUFA supplementation reduced the risk of transition at 12-months follow-up. In this study, omega-3 PUFAs also significantly reduced positive, negative, and general symptoms, and improved functioning at follow-up compared to the placebo. A subsequent study found that higher NA levels were significantly related to lower negative and positive symptoms, as well as higher global functioning in the same UHR sample at baseline [54]. However, AA and DHA levels did not exhibit any significant associations with symptoms or functioning. They also found that lower levels of NA predicted transition to psychosis in individuals who received the placebo (those who received the omega-3 PUFA supplementation were excluded due to significant treatment effects on transition). These findings remained after controlling for variables known to influence fatty acid metabolism, including age, cigarette smoking, cannabis use and antidepressant use. Notably, the observation that supplementation with omega-3 PUFAs may prevent transition to psychosis suggests that omega-3 fatty acids may offset the risk conferred by decreased levels of NA.

There is also preliminary DTI research specifically investigating the relationship between PUFA, white matter integrity and schizophrenia. Peters et al.’s [55] study of 12 patients with recent-onset schizophrenia, identified a significant positive correlation between total blood PUFA concentration and FA in the uncinate fasciculus, the white matter tract connecting the anterior temporal lobe with the orbitofrontal lobe, using a region-of-interest approach. A subsequent whole brain voxelwise analysis of 30 male patients with recent-onset schizophrenia found that lower total blood PUFA concentration was related to lower FA levels across the brain, including the corpus callosum, and bilateral parietal, occipital, temporal and frontal regions [56]. This relationship was primarily driven by AA concentration, and to a lesser extent by NA, LA and DPA concentrations. Greater severity of negative symptoms was also related to lower FA and NA concentration. These findings provide support for the notion that white matter abnormalities are related to PUFA levels in schizophrenia. Although the exact mechanism remains uncertain, it has been hypothesised that reduced PUFA levels may be related to lowered FA via inflammatory processes [56]. Specifically, AA can be transformed to proinflammatory prostaglandins and leukotrienes after being released from cell membranes, while omega-3 fatty acids can transform to anti-inflammatory factors [57]. Thus increased metabolism of omega-3 and −6 fatty acids, possible through altered immune function, may result in white matter inflammation and consequently disrupted myelination in schizophrenia. It is also possible that oxidative stress may play a role, as reduced antioxidant enzymes and increased plasma lipid peroxides have been related to lowered PUFA concentrations in first episode and never-medicated patients [58]. Further research is needed to fully unpack these potential mechanisms and gain a better understanding of biological processes that may be implicated in schizophrenia. We are currently conducting a study funded by the National Health and Medical Research Council Australia (NHMRC; APP1067040) to establish if membrane PUFA concentrations correlate with reduced white matter (WM) integrity in UHR individuals. This study investigates the clinical relevance of WM integrity and PUFA interaction in relation to illness progression in the prodromal phase of psychosis.

There are inconsistencies in the literature based on these preliminary studies that need to be addressed. Firstly, the direction of association is not consistent with that identified in healthy adolescents [45]. As mentioned above, these differences may relate to the age of samples, and there is a need for more research examining potential development effects on the relationship between blood PUFA concentration and white matter integrity in healthy individuals. Furthermore, Peters et al.’s [56] findings within the temporal lobe were reduced to trend level effects when controlling for medication and duration of illness. Therefore, it would be valuable to examine the relationship between PUFA and white matter integrity in UHR samples not confounded by such factors. It is also possible that different mechanisms underlie the relationship between blood PUFA concentrations and white matter in healthy individuals and those with schizophrenia, and future research comparing these two groups in an age-matched sample may help us better understand these differences and the potential neurobiological processes underlying schizophrenia. There are also inconsistencies in relation to the specific fatty acid that may be implicated in the disease process, and associations in PUFA alterations and symptoms may be specific to stage of illness [18]. For example, in UHR individuals, NA was found to be associated with symptoms [54], while DTI research in individuals with recent onset schizophrenia shows that AA (and to a lesser extent NA) is more strongly associated with fractional anisotropy [55]. Hence, certain PUFA alterations may be salient in the at-risk stage of illness, marking the potential to evolve into a psychotic disorder, without necessarily having a direct and causal relationship with schizophrenia [59]. This would explain why PUFA supplementation may be of benefit to very early stages of psychotic disorder (i.e., UHR individuals) and less beneficial in patients with established schizophrenia [60].

## Conclusions

There is much to be learnt about the biological underpinnings of the symptoms and deterioration of functioning characteristic of UHR individuals, and the predictive value of these markers for conversion to psychosis. There are strong international research efforts currently aimed at addressing these issues [61–63], with the hopes of developing predictive tools using imaging and other biological markers to complement currently used clinical phenotypic criteria.

Specifically addressing the issue of PUFAs and white matter integrity in UHR is the Myelin Integrity Neuroimaging (MINT) study at Orygen - The National Centre of Excellence in Youth Mental Health, Australia. This is the first study to combine neuroimaging and blood-based assessments with longitudinal clinical assessments, which will allow examination of the interaction between PUFA and white matter prior to the onset of schizophrenia. In addition, the project will examine the role of PUFAs and white matter integrity in disease progression (i.e., transition to psychosis, poorer functional outcomes) in UHR patients. It will provide us with new insight into the pathophysiology of psychosis, which will help us better understand the effectiveness of interventions altering cell membrane lipids and potentially provide the basis for development of novel treatments. Together with other cutting-edge research in this area, this study will also ultimately aid in predicting which UHR subjects will actually transition to psychosis, and their likely functional and symptomatic outcomes.

## Abbreviations

AA, arachidonic acid; DHA, docosahexaenoic acid; DPA, docosapentaenoic acid; DTI, diffusion tensor imaging; FA = fractional anisotropy; FADS, fatty-acid desaturase; fMRI, functional magnetic resonance imaging; LA, linoleic acid; MD, mean diffusivity; MRI, magnetic resonance imaging; NA, nervonic acid; PRISMA, preferred reporting items for systematic reviews and meta-analyses; PUFAs, polyunsaturated fatty acids; UHR, ultra-high risk; UHR-N, ultra-high risk negative; UHR-P, ultra-high risk positive; WM, white matter
